# Phaeomelanin matters: Redness associates with inter-individual differences in behaviour and feather corticosterone in male scops owls (*Otus scops*)

**DOI:** 10.1371/journal.pone.0241380

**Published:** 2020-11-11

**Authors:** Ángel Cruz-Miralles, Jesús M. Avilés, Olivier Chastel, Mónica Expósito-Granados, Deseada Parejo

**Affiliations:** 1 Departamento de Anatomía, Biología Celular y Zoología, Facultad de Ciencias, Universidad de Extremadura, Badajoz, España; 2 Departamento de Ecología Funcional y Evolutiva, EEZA-CSIC, La Cañada de San Urbano, Almería, España; 3 Centre d’Etudes Biologiques de Chizé, CNRS, Villiers en Bois, France; 4 Departamento de Economía y Empresa, Área de Economía Aplicada, Universidad de Almería, La Cañada de San Urbano, Almería, España; Universita degli Studi di Milano-Bicocca, ITALY

## Abstract

Individuals within populations often show consistent variation in behavioural and physiological traits which are frequently inter-correlated, potentially leading to phenotypic integration. Understanding the mechanisms behind such integration is a key task in evolutionary ecology, and melanin based colouration has been suggested to play a pivotal role. In birds, most of plumage colour variation is determined by two types of melanin, eumelanin and phaeomelanin, but the role of phaeomelanin in avian phenotype integration has been barely investigated. Here, we test for covariation between phaeomelanin-based colouration, behavioural traits (i.e. nest territoriality, aggressiveness, breath rate and parental behaviour) and corticosterone in feathers in the polymorphic scops owl *Otus scops*, a bird species in which more phaeomelanic individuals display reddish colourations. In males, we observed that reddish males took longer to return to their nests and showed higher levels of feather CORT than more greyish ones. Behaviour and feather CORT were not associated to plumage colour in females. The found associations between redness, behaviour and feather CORT in males, but not in females, might suggest the existence of a sex-specific integrated phaeomelanic phenotype in scops owls.

## Introduction

Variation in behaviour and physiology can be correlated across individuals within animal populations [1, 2], giving rise to complex phenotypes [3]. Phenotypic integration may reflect the effect of genetic, developmental or functional interactions between traits [3], and, because natural selection does not act on isolated traits, may have important consequences for the evolution of phenotypes [4]. Melanin-based colouration of teguments has been suggested to play a key role in shaping phenotypic integration [5–8].

Melanins are the pigments responsible for most non-structural brown, black and grey colour in vertebrates [9, 10], and variation is often associated with that in morphological, physiological, behavioural and life-history traits (reviewed in [5, 7, 11–13]). Birds constitute an ideal system for the study of phenotypic integration in relation to melanin-based colouration because plumage colour is frequently determined by eumelanin (responsible for grey-black colourations) and/or phaeomelanin (determining reddish-brown colour variation) [14–16]. In most birds both eumelanin and phaeomelanin are found in the same feathers [17–19]. However, most of studies do not differentiate between eumelanin and phaeomelanin, or just refer to the role of eumelanin (e.g. [5], but see [6, 20]). This might be unfortunate because if the production of eumelanin compromises the synthesis of phaeomelanin (see [5]), covariation of the two pigments with other traits would be expected to differ [21–24]. Other studies, however, report results suggesting that the synthesis of one type of melanin would not reduce or inhibit the other [6], pointing to more complex mechanisms generating melanin plumage colour [25]. In this context, it seems critical to study the role of phaeomelanin-based colours to achieve a full understanding of the role of melanins in promoting trait covariation.

Covariation between phaeomelanic colouration and other traits may arise due to the cost of production of phaeomelanin (condition-dependence hypothesis). A number of studies provide support for the idea that phaeomelanic plumage colours may function as honest signals of quality (reviewed in [26–28]). The synthesis of phaeomelanin depends on the amount of available cysteine and glutathione. Glutathione may play a critical role in anti-oxidative defence, nutrient metabolism or in regulating immune function [29]. Hence, there could be a physiological trade-off between for example anti-oxidative defence and the expression of phaeomelanin, so that only the fittest individuals would be able to display the reddest phenotypes without compromising physiology [30]. Based on these production costs, phaeomelanic colourations would have a higher potential to evolve as honest signals of quality than eumelanic ones [24]. Alternatively, covariation between phaeomelanic colouration and other traits could be due to pleiotropy, either mediated by genes involved in phaeomelanogenesis or in hormone synthesis (pleiotropy hypotheses) [31]. The melanocortin system comprises a set of five membrane receptors (*MCRs*) which regulate several functions such as melanogenesis, sexual behaviour, aggressiveness, and stress response, depending on the binding of melanocortins and the agouti signalling protein (ASIP) [5]. The binding of the melanocortins to the receptor *MC1R* (coded by melanocortin-1 receptor gene) promotes eumelanin synthesis, whereas the binding of ASIP would promote the formation of phaeomelanin [32, 33]. As melanocortins also bind to other *MCRs* than *MC1R* that regulate behaviour (e.g. *MC5R*, aggressiveness) and physiology (e.g. *MC2R*, hypothalamus-pituitary-adrenal (HPA hereafter) stress response), this may result in trait covariation [5]. Alternatively, changes in hormone secretion, hormonal affinity for carrier proteins, rates of degradation and conversion, and interaction with target tissues could potentially coordinate the co-expression of behavioural, physiological and morphological traits (hormonal pleiotropy hypothesis *sensu* [34]). In particular, corticosterone (CORT hereafter), a glucocorticoid widely investigated in birds that affects the response to stress through the activation of HPA axis, might play such a modulator role. Indeed, several studies have shown that CORT associates with behaviour [35–38] and melanin-based colouration [39–41]. However, only a handful of studies have studied covariation between phaeomelanin colourations and other phenotypic traits [42–45].

Here we study covariation between phaeomelanic plumage colour, behaviours (territoriality, aggressiveness during researcher visits and parental care) and likely correlates of stress response (breath rate and feather CORT) in male and female Eurasian scops owls (*Otus scops*) (scops owl hereafter). Breath rate is considered a reliable proxy of handling stress [46], whereas the amount of corticosterone (CORT) deposited in growing feathers provides a long-term, integrated measure of HPA activity in birds [47]. In the scops owl, feathers contain eumelanin and phaeomelanin, but most of redness variation is due to phaeomelanin [48]. Moreover, colour does not change with age in this species [49], making this an ideal system to study covariation between phaeomelanic colours and other phenotypic traits. Based on the assumption that the synthesis of phaeomelanin blocks the synthesis of eumelanin [22, 50], and, knowing that more eumelanic individuals usually display more proactive behaviours and are less stress-sensitive than less eumelanic ones (reviewed in [5]), we predict:1) that reddish individuals should exhibit more reactive behaviours (i.e. be less territorial and aggressive and show lower nest attentiveness when threatened) than greyish ones; and 2) that reddish individuals would have higher breath rate and levels of corticosterone in feathers than greyish ones. Finally, because behaviours could be subjected to sex-specific selection [34], and the hormonal pathways is likely to differ between males and females due to sexual hormones [51, 52], we predict 3) sex-specific differences in the relationships between colour, behaviour and feather CORT.

## Materials and methods

### Study system

The study was performed from 2012 to 2018 in the surroundings of the Natural park of Baza, Granada, southeast of Spain (37°18’N, 3°11’W). The area is an extensive agricultural landscape with scattered holm oaks (*Quercus ilex*) where cork-made nest-boxes have been set up to favour the reproduction of hole-nesting birds (see details in [53]).

The scops owl is a medium-sized nocturnal owl arriving from Africa into the study area in April [54, 55] and starting its reproduction throughout May [55]. Scops owls produce one clutch per year of about 4 eggs on average that are laid every second days. Females start incubating after laying the second egg, and incubation takes 24–25 days [56]. Nestling rearing takes approximately 21–29 days [54].

### Sampling procedure

Starting in the fourth week of April, nest-boxes are visited once a week until egg-laying is detected. After detection of a breeding attempt, nests are visited once more after the end of laying, and only once again just before the estimated hatching date to avoid nest desertion. After hatching, nests are visited weekly to record reproductive parameters.

Adult females were captured by hand while they were sleeping in the nest-boxes during incubation, whereas males were trapped with nest-traps while delivering food to owlets [49]. Upon capture individuals were metal ringed, sexed based on inspection of the brood patch (only present in females), and photographed for colour assignment (see below). We also collected feathers for assessment of corticosterone and measured female aggressiveness and breath rate.

### Colour characterization

Each captured individual was photographed twice: once head-on, so that head and breast plumage could be observed; and the other back on, targeting on back and wings. Photographs were taken using a digital camera (Canon EOS 1300D, Lens: EF-S 18–55 IS II) mounted on a tripod at a fixed distance of 50 cm and with a flash (aperture: 4.5, shutter speed: 1/200, ISO: 800). Owls were gently fixed with a harness inside a neutral-coloured box with the head placed next to a colour chart (X-Rite ColorChecker^®^ Passport). Photos were standardized using the Adobe^®^ Photoshop Lightroom 6 plugin and used to measure redness extension at the head, breast and wings–back. Each body part was scored among 1 to 3 points depending if they were predominantly greyish or reddish (see S1 Table in [49]. Previous results have shown that scores of the three body parts are highly correlated within individuals, and, that scores assigned by different observers on the same individual are highly repeatable [49], hence scores of the three body parts were summed to get an individual score for every bird (ranging from 3 to 9). Pigment analyses have revealed that although eumelanin is the most abundant pigment in scops owl feathers, redness variation is related to phaeomelanin: the higher the score the larger the amount of phaeomelanin in head and breast feathers [48]. Hence, colour scores were used to characterize phaeomelanic redness colouration.

### Behavioural traits

#### Male territoriality

Territoriality was measured in 35 males from 2014 to 2018 by recording behavioural responses to a simulated territorial intrusion. All trials were conducted when clutches were completed, between nightfall (mean initial time: 22:38 ± 35 minutes) and 01:00 a.m., when owls were expected to be more active. Territorial intrusions were simulated by broadcasting calls of a male scops owl with a MP3 player (takeMS “Deseo”) connected to a speaker (MOLGAR 3” 20W 4 ohm) placed under the closest tree to the target nest (at an approximate distance of 25 meters). Broadcasted records consisted of an initial 2-minutes silent track, as an acclimation period, followed with a 2-minutes track of male territorial calls followed by another 10 minutes of silence track, and a final territorial call track of 2 minutes of the same male. To avoid recognition by familiarity we extracted tracks from 3 unknown males to our population from xeno-canto (https://www.xeno-canto.org/). Territorial tracks were edited using version 2.0.3 of Audacity (R) software. MP3 compression files are widely and successfully used to imitate songs in behavioural studies of birds (e.g. [57]), so we do not expect to find effects of the MP3 format on the behavioural response in this species. Male territorial behaviour was measured using two different variables: 1) Latency of response, as the time in seconds from broadcasting to the first male hooting response; 2) Duration of response, as the time lasted in seconds from the first to the last male hooting response.

We captured nine breeding males that were deployed with radio transmitters tags (PIP Ag392 de Biotrack Ltd., Wareham, UK) the night before the simulated territorial intrusion in 2016. This allowed us to confirm whether males responding to the playback were the territory´ owners. Tags weighted between 1.10 and 1.90 g and were attached with cyanoacrylate glue onto the feathers of the back. Individuals were located by means of receivers Yaesu FT-290R II antennas (frequency range of 150 MHz). All the individuals hooting back to the simulated intrusion carried the transmitter, suggesting that they were the territory´ owners. Deployed males were re-captured the night after the simulated territorial intrusion to remove the tag without any apparent harmful effect, and, none of the nests owned by these males were abandoned after tag deployment.

#### Female aggressiveness

Female aggressiveness was measured in 45 females based on video recordings (video camera Sony DCR-SR32) made at the nest-boxes during the day, when females usually sleep. Female behaviour inside the nest-box was filmed during 20 seconds after gently opening the roof, while slowly approaching the camera to the female, and 10 seconds more while holding it in hand after its capture. Based on films females were classed as either aggressive, when they displayed any of the following behaviours: clicked the beak, hissed, swelled their body, laid on their back with claws raised, grabbed with bill or claws and/or tried to get away through that 30 seconds; or, as non-aggressive females, those feigning death in the nest and in the hand and not exhibiting any of the above behaviours.

#### Parental care

We measured parental provisioning in most scops owl nests from 2012 to 2018 (98 nests) at the beginning of the chick-rearing period (3 days after the hatching of the last egg). Parental activity was recorded at night for at least 60 minutes using infrared cameras (KPC- S500, black and white CCD camera, Esentia Systems Inc.). Upon capture, females were marked with a white Tippex spot on the head that allowed their identification in recordings. In subsequent visits to the nests and in video recordings we did not find any apparent effect of these marks on females.

From recordings, we determined: 1) latency of entering the nest-box in minutes after setting the microcamera, and, 2) adults’ feeding rates as the number of prey delivered at the nest per hour.

### Stress response

#### Breath rate

Breath rate, estimated as the number of breast movements during 30 seconds, was measured from 2015 to 2018 in 51 females and 35 males as a measure of individual response to handling stress [46]. Birds were held loosely on its back on the hand, fixing it by keeping its head between thumb and index finger and gently laying the middle finger of the other hand on the breast [46].

### Feather corticosterone

Upon capture, we collected the third covert feather of the left wing of 27 males and 43 females from 2012 to 2015 to determine CORT in feathers. Feathers were kept in hermetic plastic bags until analyses, that were performed in two batches (autumn 2014 (for samples collected from 2012 to 2014) and autumn 2015 (for samples collected in 2015)).

CORT levels in feathers were estimated by ME at the Centre d’Etudes Biologiques of Chizé, France using the method described by Bortolotti et al. [47], based on a methanol-based extraction technique. Radioimmunoassay was used to measure the CORT extracts [58], with a highly cross-reactive rabbit anti-mouse antibody from Sigma (C8784). The detection limit of the method was 0.28 ng/mL (lowest measure was 1.23 ng/mL). Although CORT in feathers was calculated in ng/mL, values were transformed to ng/mm for which feathers length (without calamus) were previously measured with a calliper to the nearest 0.1 mm.

### Statistical analysis

Analyses were performed using SAS 9.3 software (SAS Institute Inc., Cary, NC).

In a first step we estimated repeatability of behaviours for the subset of individuals with repeated samples in different years (male territoriality n = 15; breath rate, males = 15 and females = 11) by performing a linear mixed model with the trait measure as the dependent variable and the individual ID as the random intercept. This allowed us to obtain among-individual variance and within-individual variance that are used to estimate repeatability following Lessells and Boag [59]. A behaviour was considered repeatable if among-individuals variance was significantly higher than within-individuals variance, which is a reasonably assumption given low repeatability of behavioural traits (see [60]). Non-repeatable behavioural traits were not considered in subsequent analyses. We did not calculate repeatability in female aggressiveness because this feature was measured at different time in different years for a given female, potentially conditioning the test. Also, we disregarded repeatability in parental care because the number of nestlings raised by a single individual and mate identity varied among years.

Second, we run general linear models to investigate the relationships between colour scores and latency of response of males to territorial intruders, latency to enter the nest-box, feeding rates and breath rate, as dependent variables, respectively. The study year (as a categorical variable with seven levels) was also included as a fixed term. In birds, early breeders in the season are generally better quality individuals than late breeders. Hence, date of measure was introduced in the models as a further covariate to account for variation in individual quality through the season potentially affecting colouration and behaviour. Finally, brood size was included as a further covariate to control for its possible effect on parental investment.

In addition, we run generalized linear models for analysing females’ aggressiveness as a binomial dependent variable (aggressive vs non-aggressive) in relation to colour. In these models, we replicated the model structure performed above for continuous traits, but including as a covariate the hour of the day (as time in minutes until sunset) at which the response was measured to account for the fact that females were captured at different hours during the day.

Finally, we run two general linear models for analysing the relationship between CORT in feathers as dependent variable and colour scores in females and males separately. In these two models the study year was included as a fixed factor.

Standard model validation graphs [61] revealed that model assumptions of homogeneity of variance and normality of residuals were fulfilled after corticosterone in feathers, latency to return to nests and feeding rates were log-transformed.

### Ethical statement

Animal data collection complies with the current laws of Spain and the fieldwork was authorized by Consejería de Medio Ambiente y Ordenación del Territorio de la Junta de Andalucía (projects CGL2011-27561/BOS, CGL2014-56769-P and CGL2017-83503-P; license code: P06-RNM-01862). The study protocol was reviewed and approved by the ethical committee of the CSIC. Spanish law does not require ethical approval for this specific study from an International Animal Care and Use Committee (IACUC).

## Results

Latency of response of males to territorial intrusions was marginally not repeatable (r = 0.45; F_7, 9_ = 2.75, P = 0.08), but we decided to analyse it anyway. Duration of response was not repeatable (r = 0.23; F_11, 12_ = 1.59, P = 0.22), and, hence, disregarded in subsequent analyses. By contrast, breath rate was repeatable (r = 0.31; F_27, 43_ = 2.16, P = 0.01).

### Plumage colouration and behavioural traits

Latency of response to the playback of males and female aggressiveness were not explained by colouration (Tables 1 and 2).

**Table 1:** Results of the statistical models analysing male territoriality in scops owls as latency of response against an intruder in relation to plumage colouration (N = 35 individuals).

		Colour score				
Explanatory term		*β*	SE	*F*	df	*P*
Intercept		-0.66	0.52	1.27		0.21
Male colour		0.08	0.09	0.78	1, 27	0.384
Year*	2014	0.35	0.44	0.25	4, 27	0.908
	2015	0.41	0.44			
	2016	0.20	0.42			
	2017	0.27	0.36			
	2018	0.00	0.00			
Date		-0.01	0.16	0.00	1, 27	0.961
Brood size		0.00	0.13	0.00	1, 27	0.980

*The reference category for the year effect was 2018.

**Table 2:** Results of the statistical models analysing female aggressiveness in relation to plumage colouration (N = 45 individuals).

		Colour score				
Explanatory term		*β*	SE	*χ 2*	df	*P*
Intercept		-0.53	1.36	0.15	1	0.69
Female colour		-0.09	0.22	0.18	1	0.670
Hour		0.58	0.41	2.20	1	0.138
Year*	2014	2.66	1.32	7.11	4	0.130
	2015	0.87	1.21			
	2016	0.17	1.10			
	2017	1.89	1.11			
	2018	0.00	0.00			
Date		1.03	0.59	3.47	1	0.063
Brood size		0.09	0.32	0.08	1	0.775

*The reference category for the year effect was 2018.

**Fig 1:**
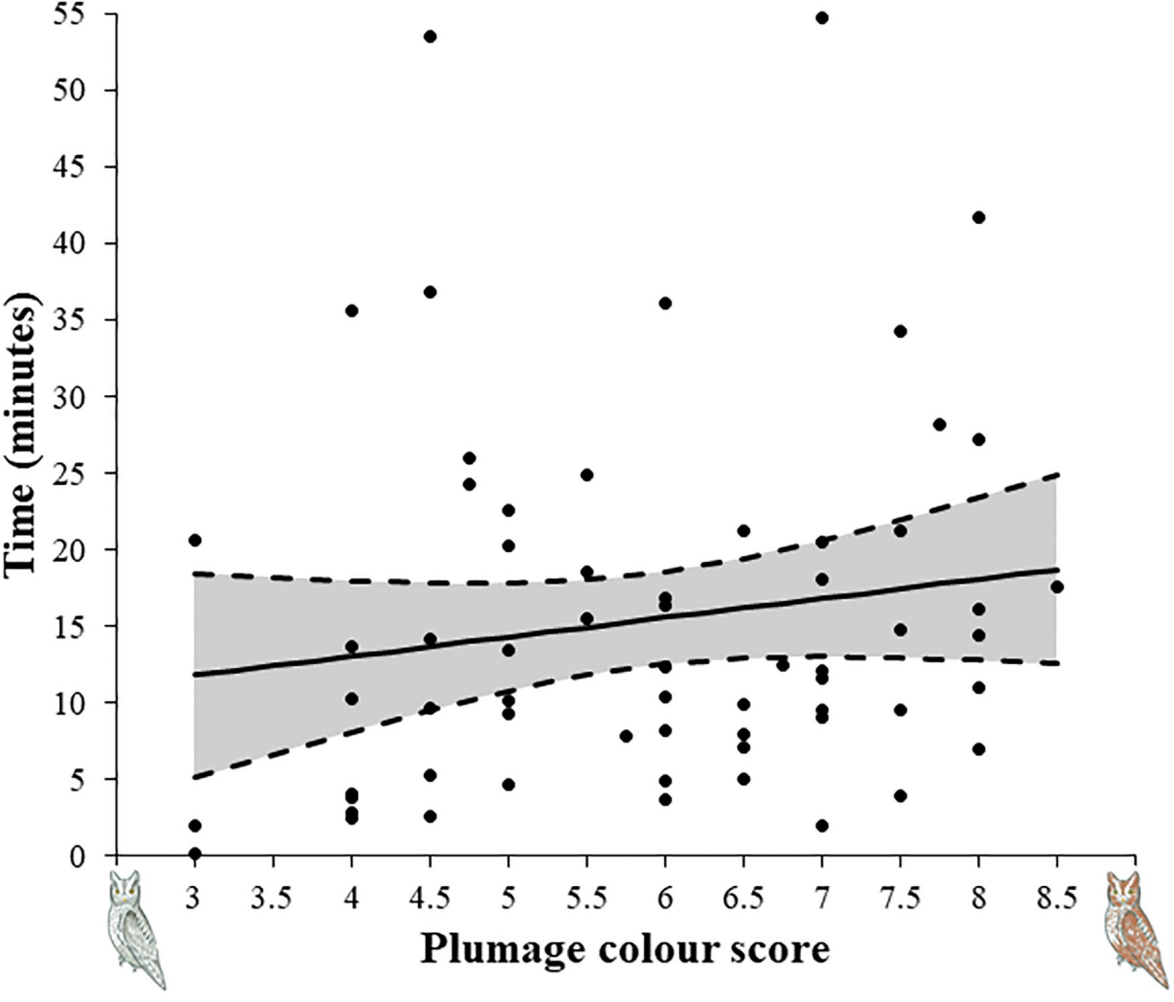
Latency to enter the nest-box of male scops owls (mean with 95% confidence interval) after setting the microcamera in relation to plumage colouration scores (N = 63).

Concerning parental behaviours, latency to enter the nest-box was related to colour in males, but not in females (Table 3). Individuals with a more reddish plumage take longer to enter the nest-box after disturbance (Fig 1).

**Table 3:** Results of statistical models analysing parental care in relation to plumage colouration.

			Males (N = 63)					Females (N = 39)				
**Dependent variable**	**Explanatory term**		***β***	**SE**	***F***	**df**	***P***	***β***	**SE**	***F***	**df**	***P***
Latency	Intercept		1.20	0.49	**2.46**		**0.02**	1.83	0.65	**2.82**		**0.008**
	Colour		0.17	0.08	**4.23**	**1, 54**	**0.045**	0.00	0.09	0.00	1, 31	0.968
	Year*	2013	0.41	0.68	0.38	5, 54	0.545	-	-	-	-	-
		2014	0.31	0.42				0.60	0.48	0.95	4, 31	0.449
		2015	0.42	0.42				0.53	0.50			
		2016	0.28	0.37				0.77	0.44			
		2017	0.04	0.35				0.67	0.45			
		2018	0.00	0.00				0.00	0.00			
	Date		0.25	0.15	2.87	1, 54	0.051	0.38	0.18	**4.65**	**1, 31**	**0.039**
	Brood size		-0.24	0.12	3.98	1, 54	0.096	-0.08	0.14	0.29	1, 31	0.594
			Males (N = 69)					Females (N = 69)				
**Dependent variable**	**Explanatory term**		***β***	**SE**	***F***	**df**	***P***	***β***	**SE**	***F***	**df**	***P***
Feeding rate	Intercept		2.15	0.37	**5.78**		**<0.0001**	1.36	0.34	**4.00**		**0.0002**
	Colour		-0.03	0.06	0.16	1, 58	0.692	-0.04	0.05	0.81	1, 59	0.373
	Year*	2012	-	-	-	-	-	-0.14	0.67	0.93	6, 59	0.482
		2013	-0.57	0.45	0.84	5, 58	0.526	-0.66	0.48			
		2014	-0.03	0.33				-0.41	0.27			
		2015	-0.45	0.32				-0.21	0.28			
		2016	-0.34	0.28				-0.19	0.26			
		2017	-0.23	0.27				0.06	0.23			
		2018	0.00	0.00				0.00	0.00			
	Date		-0.19	0.11	2.81	1, 58	0.099	0.11	0.10	1.24	1, 59	0.270
	Brood size		0.17	0.10	3.06	1, 58	0.086	0.11	0.08	1.87	1, 59	0.177

*The reference category for the year effect was 2018.

Feeding rates of female and male scops owls were not associated with plumage colour (Table 3).

### Plumage colouration and stress response

**Fig 2:**
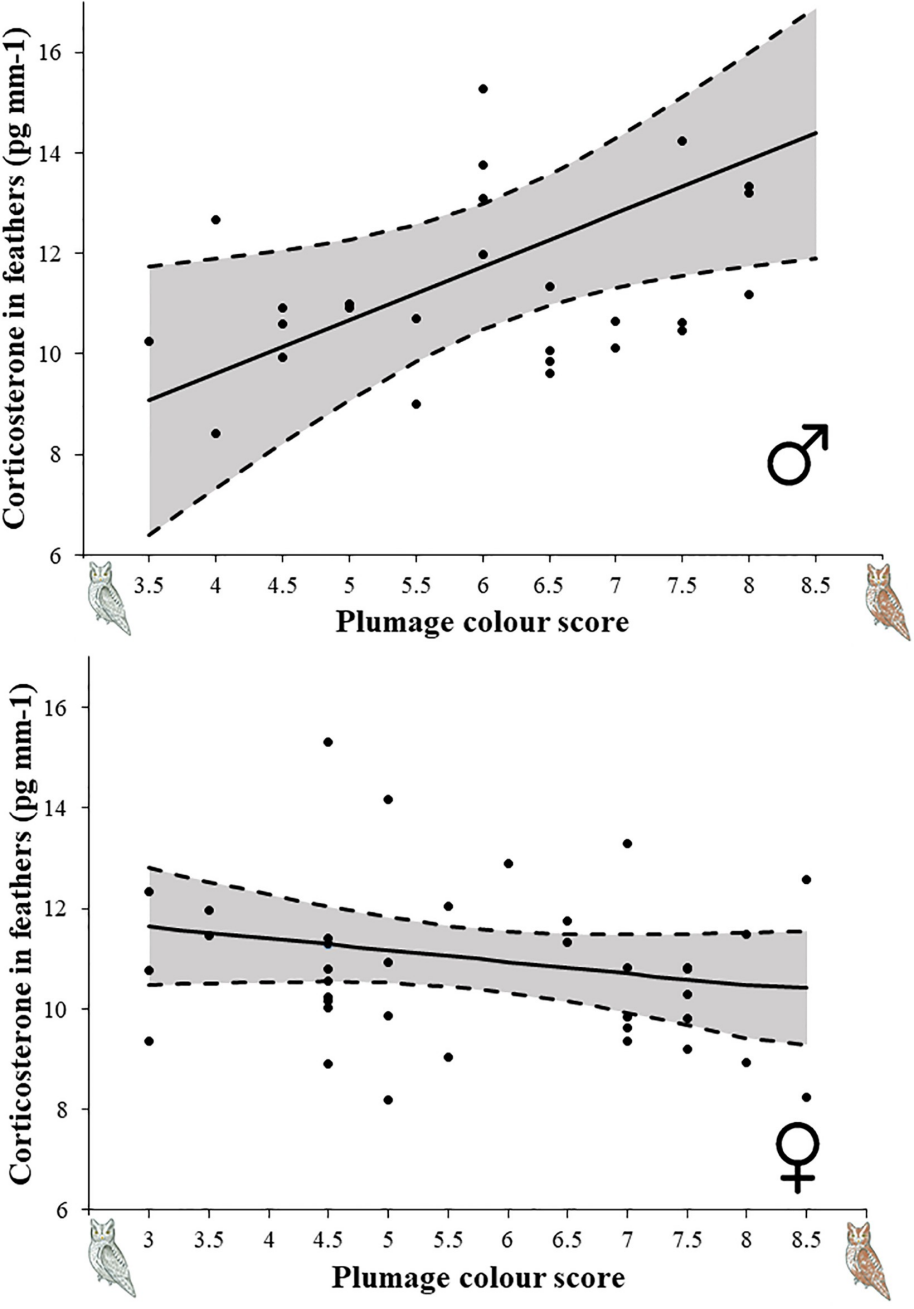
Levels of CORT in feathers (mean with 95% confidence interval) in relation to plumage colouration in male (A) and female (B) scops owls.

Breath rate was not related with plumage colouration neither in male nor in females (Table 4). However, levels of feather CORT in males, but not in females, was related to plumage colouration (Table 4). Reddish males had higher levels of feather CORT than greyish ones (Fig 2).

**Table 4:** Results of GLMs to analyse breath rate and CORT in feathers in relation to plumage colouration in males and females scops owls.

			Males (N = 35)					Females (N = 52)				
**Dependent variable**	**Explanatory term**		***β***	**SE**	***F***	**df**	***P***	***β***	**SE**	***F***	**df**	***P***
Breath rate	Intercept		0.49	0.69	0.70		0.489	0.17	0.60	0.28		0.777
	Colour		-0.02	0.12	0.02	1, 27	0.894	-0.01	0.10	0.02	1, 43	0.895
	Hour		-0.32	0.27	1.48	1, 27	0.234	-0.39	0.16	**5.95**	**1, 43**	**0.019**
	Year*	2015	-0.56	0.57	1.56	3, 27	0.222	0.22	0.49	0.20	3, 43	0.895
		2016	-1.21	0.67				0.00	0.45			
		2017	-0.61	0.41				-0.16	0.41			
		2018	0.00	0.00				0.00	0.00			
	Date		0.22	0.20	1.25	1, 27	0.274	-0.09	0.21	0.20	1, 43	0.660
	Brood size		-0.21	0.17	1.46	1, 27	0.238	-0.21	0.13	2.60	1, 43	0.114
			Males (N = 28)					Females (N = 42)				
**Dependent variable**	**Explanatory term**		***β***	**SE**	***F***	**df**	***P***	***β***	**SE**	***F***	**df**	***P***
CORT in feathers	Intercept		0.89	0.09	**9.77**		**<0.0001**	1.14	0.05	**23.62**		**<0.0001**
	Colour		0.03	0.01	4.13	1, 22	0.055	-0.01	0.01	2.27	1, 36	0.140
	Year*	2012	-0.07	0.10	0.34	3, 22	0.794	-0.06	0.03	1.70	3, 36	0.184
		2013	0.00	0.05				-0.04	0.03			
		2014	0.02	0.04				-0.06	0.03			
		2015	0.00	0.00				0.00	0.00			

*The reference categories for the year effect were 2018 for the models on breath rate, and 2015 for the models on CORT in feathers, respectively.

## Discussion

Our results tentatively support the existence of a phaeomelanic syndrome encompassing a suite of correlated behavioural and physiological traits in male scops owls. First, feather CORT differed with redness so that the reddish males had higher CORT values than grey ones. On the other hand, reddish males required more time to resume feeding than greyish ones after being disturbed in their nests. Colour variation, however, did not associate with behaviour and feather CORT in females. Below we discuss the most likely explanations for the causes and sense of patterns based on current knowledge about the role of phaeomelanin and corticosterone in determining phenotypic variation.

One possibility to explain the association between male colouration and feeding activity would be provided by the melanocortin hypothesis [5]. Membrane receptors (*MCRs*) involved in melanogenesis could control sexual behaviour, aggressiveness, and stress response [5]. However, we recently have found that variation in the coding sequence of the *MC1R* does not explain variation in redness in this species [48]. Nonetheless, more than 150 genes involved in colour expression in animals have been identified, and many of them could be involved in phaeomelanins synthesis [21]. Hence, we can only discard a pleiotropic effect of *MC1R* but not of other genes potentially involved in melanogenesis such as *MIFT*, *ASIP*, *TYR*, *SLC45A2* and *TYRP1* (e.g. [62–65]).

Alternatively, the found association between colour and behaviour in males could be mediated by corticosterone [31]. We found that reddish males have higher levels of CORT in feathers than more greyish ones, suggesting that individuals differing in phaeomelanic colour may have different sensitivity to stress during the moult period. However, breathing rate (i.e. a proxy of handling stress) was unrelated with phaeomelanic colour in males, a pattern that was also found in rock pigeons (*Columba livia*) in relation to eumelanic colouration [66]. Previous studies have found that breath rate might also be related to personality and risk taking [46], and, therefore, we cannot exclude the possibility that breathing rate was reflecting differences in personality rather than in stress response. Alternatively, given that feather CORT is likely to reflect the accumulated stress during the time of feather growth [47], the pattern could arise because males differing in colouration have faced different stressors during moult time (i.e. moulting at different time or places or following a different moult pattern (e.g. [67]). A particularly fruitful field of future research which may help to disentangle these possibilities would be to study how individuals differing in phaeomelanic colour use space and time and forage outside the breeding season by deploying GPS devices in combination with the study of feather CORT.

The relationships between phaeomelanic colours and other functional traits seemed to differ between sexes, a pattern that has already been reported for eumelanic colourations (e.g. common buzzard *Buteo buteo*; [68]; marsh harrier *Circus aeruginosus*; [69]; masked boby *Sula dactylatra* [6]). Sexual differences in covariation could arrive from variation in the relative role of eumelanin versus phaeomelanin influencing trait expression in the two sexes. However, pigment analyses have revealed no sexual differences in the relative importance of the two melanin pigments in scops owls [48]. In females, however, feather CORT was not associated with colouration. Differences between females and males may be due to the role of sexual hormones. Indeed, levels of testosterone have been reported to negatively correlate with CORT [51, 52], whereas oestrogens enhance glucocorticoids responses [70]. Also, sexes could show differential sensitivity to hormones in the brain mediated by the abundance of androgen receptors, aromatase or oestrogens [71, 72]. Alternatively, given that patterns found in males are based on feather CORT, which likely reflects stress during feather development, it could be argued that males and females are not under the same stressors when they moult feathers because they moult at different places, or that they do not moult at the same time.

Our results would support expectations from a key role of mechanisms involved in phaeomelanin synthesis in determining traits associations in male scops owls. Whatever the physiological mechanism behind, the expression of eumelanin and phaeomelanin colours are expected to be inversely related to other functional traits [21, 22, 24]. So far, covariation between eumelanic colours and behaviour has been widely investigated in birds (reviewed in [5]), but only recently a few studies have considered covariation between phaeomelanic colours and behaviour, showing contradictory results [20, 42, 43, 50]. First, Van den Brink and co-workers did not detect any relationship between behaviour and the reddish phaeomelanic colouration in both the Eurasian kestrel *Falco tinnunculus* [43] and the barn owl *Tyto alba* [50]. However, redness was positively associated with antipredator behaviour in tawny owls *Strix aluco* [42] and barn swallows *Hirundo rustica* [20]. As expected, our results show that reddish scops owl males show more reactive behaviour than more grey ones. Differences among studies might be due to the different relative role of eumelanin versus phaeomelanin in determining colouration and behaviour in different species, a possibility that merits further investigation.

Concerning corticosterone levels, a number of studies have previously investigated their association with eumelanin-based traits in birds and shown that in general darker eumelanic individuals have lower stress-induced CORT levels ([39, 40], see however [6]). The association of phaeomelanin and the stress response, however, remains elusive. Some studies do not find a significant association between phaeomelanin colourations and circulating basal or stress-induced CORT (North America barn swallows *Hirundo rustica erythrogaster*, [23] or feather CORT (yellow warblers *Setophaga petechia*, [73] levels. However, another study in barn swallows shows that darker phaeomelanic males had higher baseline and stress-induced levels of circulating CORT [74]. In agreement with expectation from a contrary role of eumelanin and phaeomelanin, we found that reddish scops owl males showed higher levels of feather CORT which may suggest that reddish individuals would be less prepared to cope with stress during moulting. Nevertheless, as above stressed, it is also possible that variation in phaeomelanic colour determined first the behaviour and/or moulting pattern [67], and, as a by-product, the stress response.

### Limitations of the study

Our study has some weaknesses worth mentioning that may affect the strength of our conclusions. First, due to logistic issues during data collection, we did not study multiple trait covariation, but independent pair-wise covariation. This limits our potential to conclude about complete phenotype integration in scops owls, and it remains to be studied if the found patterns in relation to colour morphs form part of a higher level of integration. Second, given huge differences in reproductive behaviours between sexes, we could not measure the same behaviours in males and females. Future studies aiming to investigate sex-specific phenotypic integration should ideally target non-reproductive periods, which are less likely affected by sex.

## Conclusions

The found relationships between phaeomelanin-based colour, behaviour and feather CORT in males might suggest the existence of an integrated phaeomelanic phenotype in scops owls. This is one of the first studies showing a role of phaeomelanin underlying the covariation between melanic colouration and other phenotypic traits, urging for more investigation into the genetic basis linking behaviour and stress-related hormones with the production of this pigment. Finally, although we have found sex-specific covariation among functional traits, our work identifies practical difficulties to study phenotype integration in the reproductive period, where selective pressures for the functional association among behaviour, endocrine profile, and colouration might differ between sexes.

## Supporting information

S1 Table Number of individuals, grouped by year and sex, in which the different behavioural traits and feather corticosterone were measured. (DOCX)
