# Supplementary material for: Phaeomelanin matters: Redness associates with inter-individual differences in behaviour and feather corticosterone in male scops owls (Otus scops)
Source: PLoS One. 2020 Nov 11;15(11):e0241380. doi: 10.1371/journal.pone.0241380 (PMC7657523; doi:10.1371/journal.pone.0241380)
Supplement: S1 Table — (DOCX) [file pone.0241380.s001.docx]

**S1 Table. Number of individuals, grouped by year and sex, in which the different behavioural traits and feather CORT were measured**

|  | **Years** | | | | | | | | | | | | | | | | | | | | |  | | |  | | |
| --- | --- | --- | --- | --- | --- | --- | --- | --- | --- | --- | --- | --- | --- | --- | --- | --- | --- | --- | --- | --- | --- | --- | --- | --- | --- | --- | --- |
|  | **2012** | | **2013** | | | **2014** | | | **2015** | | | **2016** | | | **2017** | | | **2018** | | | **Total** | | | | |  |  |
|  | **Females** | **Males** | | **Females** | **Males** | | **Females** | **Males** | | **Females** | **Males** | | **Females** | **Males** | | **Females** | **Males** | | **Females** | **Males** | | | **Females** | **Males** | | |  |
| **Territoriality** | - | - | | - | - | | - | 6 | | - | 6 | | - | 6 | | - | 11 | | - | 6 | | | - | 35 | | |  |
| **Aggressiveness** | - | - | | - | - | | 6 | - | | 7 | - | | 9 | - | | 12 | - | | 11 | - | | | 45 | - | | |  |
| **Latency** | - | - | | - | 2 | | 6 | 9 | | 9 | 11 | | 7 | 10 | | 9 | 16 | | 8 | 15 | | | 39 | 63 | | |  |
| **Feeding rate** | 1 | - | | 2 | 3 | | 11 | 9 | | 12 | 12 | | 11 | 11 | | 17 | 17 | | 15 | 15 | | | 69 | 67 | | |  |
| **Breath rate** | - | - | | - | - | | - | - | | 11 | 6 | | 12 | 6 | | 15 | 16 | | 13 | 7 | | | 51 | 35 | | |  |
| **Corticosterone in feathers** | 8 | 1 | | 13 | 5 | | 12 | 10 | | 104 | 11 | | - | - | | - | - | | - | - | | | 43 | 27 | | |  |
